# Practical Notes and Formulæ

**Published:** 1885-02-20

**Authors:** 


### PRACTICAL NOTES AND FORMULÆ.

**Pleasant Antiperiodic.**—A pleasant antiperiodic, especially for children, is as follows :

℞. Cinchonia alkaloid.............................grs. xvj,
Sodæ bicarb......................................grs. v,
Aquæ menth. pip..................................℥ ss,
Syrup simpli.....................................℥ jss. M.

Sig. : One teaspoonful, for a child about one year old.

Directions for proper preparing : Dissolve the soda in the peppermint water ; put the cinchona in a small mortar and add, gradually, by constant trituration, syrup to about an ounce ; add to the soda solution, and pour into a two-ounce bottle ; then, by triturating the remaining half-ounce of syrup in the mortar, all adhering cinchona will be thus incorporated, and the whole makes two ounces.

For an irritable state of the stomach, may be added :

℞. Ether.............................................gtt. j,

Syr. ipecac.........................................ʒ j,

Alcohol or spts. lavender comp......................ʒ j.

—*Medical World.*

**Tetter Ointment.**—The following (says the Druggists' Circular) may be of service :

Powder and mix 2 drachms calomel, 1 drachm sugar of lead, and ½ drachm red precipitate. Make 42 grains of this mixture into an ointment with 2 drachms of lard or simple cerate.

**Compound Hydrastis Ointment.**—Dr. George W. Sloane contributes the following formula to Lillard's "Practical Hints and Formulas" :

℞. Glycerin..........................................℔ 1

Spermaceti..........................................℥ 6

White wax...........................................℥ 2

Oxide zinc..........................................℥ 3

Hydrastine sulph....................................℥ ½

Morphine sulph......................................grs. 24

Rose geranium.......................................gtts. 20

Put the glycerin into a porcelain dish, and place upon the water-bath until warm. Having placed the zinc, hydrastine, and morphine into a suitable mortar, rub into an intimate powder without much pressure. When the glycerin is warm, add about one-third of it to the mortar containing the dry articles. To the balance of the glycerin add the wax and spermaceti. When melted, add the contents of the mortar and the oil rose geranium, and stir constantly and rapidly. If carefully done there will be no granulation.—*National Druggist.*

**Arnicated Glycerole of Cantharides for the Hair.**—

℞. Ammonium carbonate............................ʒ i

Ammonium hydrochlorate.........................ʒ i

Salicylic acid.....................................grs. 20

Water, q. s. to make...............................℥ 8

Dissolve, and add the following, previously mixed:

Tincture arnica...................................fl. ℥ 2

Tincture cantharides...............................fl. ℥ 2

Alcohol.............................................fl. ℥ 2

Glycerin..........................................℥ i

Cologne water.....................................℥ i

Filter through magnesium carbonate until clear.

This preparation is said to stimulate the growth of the hair, rendering it, at the same time, soft and glossy in appearance. It should be used in small quantities night and morning, being well rubbed into the roots of the hair with the ends of the fingers. Owing to the cantharides, discretion must be employed when used on women and children.— *Western Druggist.*

**Angostura Bitters.**—The following formul was communicated some years ago by a subscriber to this journal, and is the best one of which we know. It has been used by many druggists, and extensively copied by other journals:

℞. True Angostura bark.......................4 ounces.
Chamomile flowers..........................1 ounce.
Cardamon seeds.............................2 drachms.
Cinnamon....................................2 drachms.
Orange peel.................................1 ounce.
Raisins......................................1 pound.
Diluted alcohol..............................2½ gallons.

Macerate for one month, then press and filter.—*Ib.*

**Compound Syrup of Horehound.**—King's American Dispensatory contains the following formula, which we have modified slightly as regards manipulations:

℞. Red-root bark (*Ceanothus americanus*).......
Elecampane root (*Inula Helenium*)..........
Spikenard root (*Aralia racemosa*)...........
Comfrey root (*Symphytum officinale*)........
Wild cherry bark (*Prunus serotina*).........
Horehound, leaves and top (*Marrubium vulgare*), each............................1 pound.
Bloodroot (*Sanguinaria canadensis*),........ ½ "
Granulated sugar.............................24 pounds.

Grind to a medium powder; mix well; moisten with 76 per cent. alcohol; pack in a suitable percolator; cover with menstrum, and macerate 3 days. Then draw off three pints of percolate, and set it aside to be used later. Continue the percolation with water. When all the alcohol has been displaced, take this second percolate and deprive it of alcohol by distillation, or by evaporation. Continue the percolation with water until the liquid is nearly tasteless. Mix these two aqueous liquids, add the sugar to them, heat to boiling, remove scum, and evaporate to 21 pints. When cold, add the reserved 3 pints of alcoholic percolate.

Dose, half fluid ounce three or four times a day. This is highly recommended as a cough medicine.—*Druggists' Circular*.

**Application for Diptheria.**—The following will be found a most useful formula :

℞. Liq. ferri sub. sulph. . . . . . . . . . . . . . . . . . . . . . . . . ʒ iv,

Acid carbol. . . . . . . . . . . . . . . . . . . . . . . . . . . . ʒ j,

Sodæ sulph. . . . . . . . . . . . . . . . . . . . . . . . . . . . ʒ iij,

Glycerini . . . . . . . . . . . . . . . . . . . . . . . . . . . . ℥ ij,

Aquæ, ad. . . . . . . . . . . . . . . . . . . . . . . . . . . . ℥ iv.

M. Sig. : Apply by means of a brush or swab every two or three hours.—*Medical Digest*.

**For Nervous Prostration.**—The Medical Summary furnishes the following prescription :

℞. Phosphoric acid, dil. . . . . . . . . . . . . . . . . . . . . . . . ℥ j,

Elix. calisaya . . . . . . . . . . . . . . . . . . . . . . . . . . ℥ iv,

Elix. val. ammon. . . . . . . . . . . . . . . . . . . . . . . . . ℥ ij,

Glycerine . . . . . . . . . . . . . . . . . . . . . . . . . . . . ℥ iij,

Sherry wine, ad. . . . . . . . . . . . . . . . . . . . . . . . . Oj.

M. S. : One to two tablespoonfuls three or four times a day.

**Rheumatism.**—Try the following for a local treatment in rheumatism :

℞. Chloroform . . . . . . . . . . . . . . . . . . . . . . . . . . . ℥ i,

Gum camphor . . . . . . . . . . . . . . . . . . . . . . . . . . . ℥ ½,

Ol. olivæ. . . . . . . . . . . . . . . . . . . . . . . . . . . . ℥ ii,

Aquæ amm . . . . . . . . . . . . . . . . . . . . . . . . . . . . ℥ i,

Tinct. opii . . . . . . . . . . . . . . . . . . . . . . . . . . . ℥ i.

M. Sig. : Shake, and use as a liniment.

**Treatment of Boils.**—Dr. John Aulde, following the suggestion of Dr. Sidney Ringor, has met with the most satisfactory results by adopting the following plan. The diet is to be regulated, and if constipation exists a teaspoonful of magnesia sulp. in a glass of cold water should be taken every morning before breakfast :

℞. Calcii sulphidi . . . . . . . . . . . . . . . . . . . . . . . . 3 grs.
Sacch. lactis . . . . . . . . . . . . . . . . . . . . . . . . . 30 grs.

Misce bene et div. in chart, No. xxx.

Sig. : Five powders daily, at intervals, between meals.

By this method, beginning boils will be aborted, and those far enough advanced to threaten a siege of several weeks, and successive crops, will soften and heal in such short time that the patient will be surprised at the result. When they can be obtained, granules, containing one-tenth grain, are to be preferred to the powders. The urine should be examined for sugar, as boils and diabetes often go together.—*Summary*.

